# Radiographic analysis of dynamic lumbar motion during the five-repetition sit-to-stand test in degenerative lumbar spondylolisthesis

**DOI:** 10.1186/s12891-022-05761-4

**Published:** 2022-08-22

**Authors:** Jiang Jiang, Jun Hu, Hai-ping Cai, Lei Niu, Meng-long Zheng, Xi Chen, Wen‑zhi Zhang

**Affiliations:** 1 Department of Orthopedics, The Affiliated Provincial Hospital of Anhui Medical University, Hefei, China; 2 Department of Orthopedics, The First Affiliated Hospital of USTC, Division of Life Sciences and Medicine, University of Science and Technology of China, Hefei, China; 3 Department of Radiology, The First Affiliated Hospital of USTC, Division of Life Sciences and Medicine, University of Science and Technology of China, Hefei, China; 4 Hefei, China

**Keywords:** Low back pain, 5R-STS test, Sit to stand, Lumbar spondylolisthesis, Objective functional impairment

## Abstract

**Background:**

To investigate the mechanisms of low back pain triggered by the five-repetition sit-to-stand test (5R-STS test) in degenerative lumbar spondylolisthesis (DLS) from radiographic perspective, as well as to determine the most useful diagnostic modalities in the evaluation of segmental instability.

**Methods:**

We retrospectively performed a study of 78 patients (23 men and 55 women) with symptomatic DLS at L4/5 in our institution between April 2020 and December 2021. Each patient was assessed by using the 5R-STS test and received a series of radiographs including the upright standing, normal sitting, standing flexion–extension radiographs, and supine sagittal MR images. Enrolled patients were divided into two groups based on the 5R-STS test score: severe group and mild group. Translational and angular motion was determined by comparing normal sitting radiograph (N) with upright standing radiograph (U) (Combined, NU), flexion/extension radiographs (FE) as well as normal sitting radiograph (N) with a supine sagittal MR image (sMR) (Combined, N-sMR).

**Results:**

Overall, 78 patients were enrolled, and there were 31(39.7%) patients in group S and 47(60.3%) patients in group M, with an average age of 60.7 ± 8.4 years. The normal sitting radiograph demonstrated the maximum slip percentage (SP) and the highest kyphotic angle both in group S and group M. Compared with group M, group S revealed significantly higher SP in the normal sitting position (24.1 vs 19.6; *p* = 0.002). The lumbar slip angular in group S with a sitting position was significantly higher than that in group M (-5.2 vs -1.3; *p* < 0.001). All patients in group S had objective functional impairment (OFI) and 28 patients of them were diagnosed with lumbar instability by using the combination of normal sitting radiograph (N) and supine sagittal MR image (sMR) (Combined, N-sMR).

**Conclusion:**

DLS patients with positive sign of the 5R-STS test is a distinct subgroup associated with lumbar instability. The modality of the combination of normal sitting radiograph (N) and supine sagittal MR image (sMR) had a significant advantage in terms of the ability to identify segmental instability.

## Introduction

Degenerative lumbar spondylolisthesis (DLS) is a common progressive and discogenic disease that occurs in elderly people and develops with age [1]. The coexisting conditions of disc degeneration, facet joint hypertrophy, ligament thickening and segmental instability in the spinal motion unit often result in low back pain and functional impairment and sometimes neurological deficits in a number of patients [2, 3]. Usually, some patients who present with low back pain and disability related to DLS will experience pain relief and functional recovery after conservative treatment. However, another portion of patients still fail to respond to conservative treatments and may opt for surgical intervention [4, 5]. For patients who suffer from severe symptoms or progressive neurological deficits, although there remains some debate in determining the optimal surgical method in certain clinical situations, a surgical procedure of decompression with or without additional instrumentation of the involved spinal segments has proven to be highly effective [4, 6].

In clinical practice, the decisions for surgical treatment of DLS are predominantly based on back and/or leg pain, functional disability and impaired health-related quality of life in the simultaneous presence of corresponding radiological imaging findings [7]. Other considerations, such as age, concomitant diseases, symptom duration, surgical efficacy and the individual expected outcomes, may also play a role in the surgical decision-making process [8]. Therefore, accurate assessment of objective functional impairment (OFI), rather than the patients’ subjective perception of his or her pain and disability, becomes paramount when considering patients for surgery as well as when providing counseling regarding surgical outcomes and expectations [2, 9]. For the considerations mentioned above, several tests, such as the timed up-and-go test (TUG), 6-min walk test (6MWT) and the five-repetition sit-to-stand test (5R-STS test), have been proposed and validated in the assessment of OFI in patients with DLS and provide new adjunctive dimensions in helping clinical decision-making [9, 10].

Low back pain is the main cause of OFI in elderly patients with DLS [7]. Low back pain has multifactorial causes, including inflammatory diseases, facet arthropathy, ligamentum flavum thickening and discogenic pain, but the primary reason is lumbar instability [11–13]. Clinical manifestations of low back pain are also varied in many aspects, including potential triggers, onset sites and responses to treatments [9]. Spinal movement-triggered pain is one of the characteristics of low back pain [14]. Some patients with low back pain are obviously aggravated when they change body posture [9, 15]. In our previous study, we identified a subset of patients with low back pain due to lateral displacement during spinal bending motion. In addition, other patients suffer from low back pain during sit-to-stand movements [9, 15, 16]. Low back pain triggered by the movement of sitting to standing has a substantial impact on patients’ daily lives and results in OFI [7, 10, 16]. Several studies have employed the 5R-STS test, which is a quick and convenient standardized test that can objectively assess functional impairment in patients with degenerative pathologies of the lumbar spine [16, 17]. However, the 5R-STS test does not fully explain the pathogenesis of low back pain accompanied by postural change. Thus, the aim of our study was to investigate the mechanisms of low back pain triggered by the 5R-STS test in degenerative lumbar spinal conditions from a radiographic perspective.

## Materials and methods

### Patients

From April 2020 through December 2021, we retrospectively reviewed the records of patients in our institution. The patients enrolled in this study had to meet the following criteria: (1) monosegment L4/5 instability; (2) low-grade lumbar spondylolisthesis; and (3) a complete set of radiological examinations, including normal sitting, standing upright, flexion, extension radiographs, and supine sagittal MR images. Patients were excluded for any of the following criteria: (1) Cobb angle > 10°; (2) spondylolisthesis at L4/L5 of grade III and above; (3) segment instability at any other level in the lumbar spine; or (4) history of prior spinal trauma, surgery, infection, or fracture of the pelvis or lower limbs.

### Evolution of baseline characteristics

Baseline characteristics included patient information (age, sex and BMI), patient-reported outcomes and basic radiographic parameters [18]. The patient-reported outcomes were evaluated by the Oswestry Disability Index (ODI) and the visual analog scale (VAS) of leg and back pain [19, 20]. In addition, pain characteristics were evaluated through questionnaires consisting of the following items: pain on bending over to tie shoes, pain on climbing stairs, pain on rising from a chair, pain on walking some distance, pain on prolonged standing and pain on daily physical activities.

### 5R-STS test

**Fig. 1:**
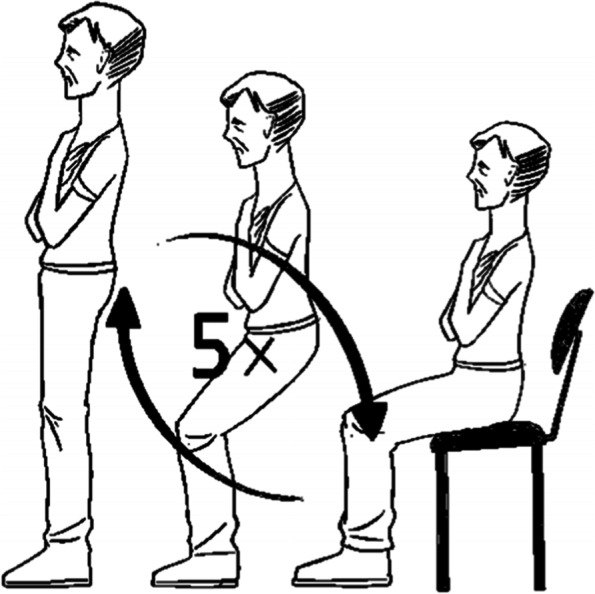
Scheme of the 5R-STS test

The 5R-STS test was conducted by asking enrolled patients to sit down on an armless chair of standard height (48 cm) and with a straight back firmly placed against a wall. During the measurement, seated patients were asked to fold their arms crossed across their chest and to keep their feet flat on the floor. To become familiarized with the maneuver, participants were then instructed to fully stand and sit down only once without using their upper limbs. The test was abandoned if the participant could not complete the initial movement or required assistance. Otherwise, participants were asked to stand up fully and then sit down landing firmly five times as fast as possible while keeping their arms folded across their chests (Fig. 1). Timing with a stopwatch was started from the initial command to the completed fifth stand. This time taken was recorded as the participant’s score. Participants were given a score of 30 s if they were unable to complete the test in 30 s or at all. According to the patients’ 5R-STS test time in seconds, participants who completed the test in less than 10.4 s were considered to have no relevant objective functional impairment [16, 17].

### Radiographic evaluation

Basic radiographic parameters on X-rays and MRI images include slip percentage, slip angle, and anterior and posterior disc height [14]. To reduce the measurement errors, enrolled patients were measured twice by a team of experienced technologists, and the mean values were used for statistical analysis. The slip percentage (SP) of the translated vertebra was measured as the ratio of the measured slip distance to the length of the upper endplate of L5. The slip angle (SA) of the spondylolisthesis level was calculated as the angle between the L4 lower endplate and the L5 upper endplate [2]. For the measurement of lumbar spine angulation, lordosis was defined as a positive angle, while kyphosis was defined as a negative angle. Translational motion and angular motion were calculated by taking the absolute values of the difference between the radiographs at the two positions. Translational motion between different positions ≥ 8% or intervertebral angular rotation between positions ≥ 10° was regarded as segmental instability [2, 12, 21].

### Study groups

The enrolled patients were divided into two groups according to their 5R-STS test score: the severe group (Group S, ≥ 22.0 s) and the mild group (Group M, < 22.0 s). Group S included patients with test times greater than 22.0 s as having severe functional impairment. Group M included patients with test times less than 22.0 s as having mild functional impairment [16].

### Statistical analysis

All data were analyzed using SPSS software (version 17.0, SPSS, Chicago, IL, USA) and are presented as the mean ± standard deviation. A *p* value < 0.05 was considered significant. Baseline characteristics between Group S and Group M subjects were compared using Welch’s t test for unequal variances for continuous variables and the chi-squared test for categorical variables.

## Result

### Baseline characteristics analysis

The baseline characteristics of the study cohort are shown in Table 1. In total, 78 patients were enrolled in the study, including 23 males (29.5%) and 55 females (70.5%). The average age was 60.7 ± 8.4 years (range: 36–87 years). Thirty-one (39.7%) patients were assigned to Group S. There was no significant difference in age, sex or BMI between Group S and Group M. The patients in Group S had significantly higher 5R-STS test times than those in Group M (24.9 ± 4.7 vs. 11.9 ± 3.2, *p* < 0.001). The patients in Group S all had OFIs, while in Group M as many as 38.3% (18 out of 47) of patients had OFIs (Table 1).

**Table 1:** Patient’s baseline characteristics evolution

Characteristic	Group S(*n* = 31)	Group M(*n* = 47)	*P* Value
Sex (M/F)	7/24	16/31	0.28
Age (years)	60.6 ± 7.4	58.8 ± 9.8	0.35
Height,cm	171.1 ± 4.9	169.8 ± 6.6	0.32
Weight,kg	68.5 ± 4.3	67.4 ± 4.2	0.26
BMI,kg/m	25.3 ± 2.6	24.5 ± 2.9	0.21
5STS (s)	24.9 ± 4.7	11.9 ± 3.2	< 0.001^*^
OFI(yes)	31	18	< 0.001^*^

^*^Statistically significant between the group S and group M

### Patient-reported outcomes and pain characteristics evaluations

In terms of patient-reported outcomes, Group S had worse low back pain (7.6 ± 1.0 vs. 5.2 ± 0.9, *P* < 0.001) and ODI scores (47.3 ± 6.2 vs. 34.1 ± 3.1, *P* < 0.001), but there was no significant difference in the VAS scores of leg pain between the two groups. Further analysis of the pain characteristics showed that Group S was characterized by pain when performing physical activities, bending over to tie shoes, climbing stairs, and rising from a chair (all *p* values < 0.05) (Table 2).

**Table 2:** Evaluation of patient-reported outcomes and pain characteristics

Variable/Groups	group S	group M	*P* Value
VAS (Back)	7.6 ± 1.0	5.2 ± 0.9	< 0.001^*^
VAS (Leg)	5.7 ± 1.2	5.9 ± 1.1	0.25
ODI	47.3 ± 6.2	34.1 ± 3.1	< 0.001^*^
Pain on bending over to tie shoes			
Yes	27(87.1%)	11 (23.4%)	< 0.001^*^
No	4(12.9%)	36 (76.6%)	
Pain on climbing stairs			
Yes	24 (77.4%)	20(42.6%)	0.02^*^
No	7(22.6%)	27(57.4%)	
Pain on rising from a chair			
Yes	23 (74.2%)	19(40.4%)	0.03^*^
No	8 (25.8%)	28(59.6%)	
Pain on walking some distance			
Yes	18(58.1%)	21(44.7%)	0.25
No	13(41.9%)	26(55.3%)	
Pain on prolong standing			
Yes	21 (67.7%)	23(48.9%)	0.10
No	10(32.3%)	24(51.1%)	
Pain on daily physical activities			
Yes	21(67.7%)	16(34.0%)	0.004^*^
No	10(32.3%)	31(66.0%)	

*VAS* indicates visual analogue scale, *ODI* indicates oswestry disability index

^*^Statistically significant between the group S and group M

### Comparison of translational motion and angular motion between Group S and Group M

**Fig. 2:**
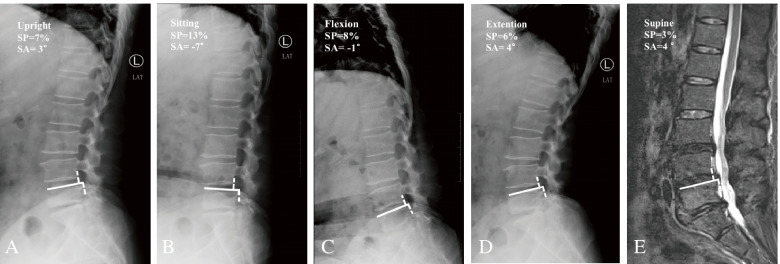
**A** 61-year-old female with L4/5 degenerative lumbar spondylolisthesis. The normal sitting radiograph showing a kyphotic slip angle at the involved segment **B**. The N-sMR analysis (**B**, **E**) demonstrated higher segmental mobility than FE (**C**, **D**) (10% vs. 2%)

In both Group S and Group M, the sitting radiograph revealed the maximum SP, whereas supine sagittal MR images revealed the minimum SP (Fig. 2). Compared with Group M, Group S demonstrated significantly higher SP in the sitting position (24.1 ± 6.3 vs. 19.6 ± 5.7; *p* = 0.002*) and extension position (15.7 ± 3.9 vs. 11.9 ± 4.1; *p* < 0.001), whereas there was no significant difference in the upright, flexion, extension and supine MRI positions (all *p* values > 0.05). In terms of segmental instability, the translation motion was significantly higher in Group S than in Group M in the N-sMR analysis (13.7 ± 4.3 vs. 8.1 ± 3.7; *p* < 0.001). However, there was no significant difference in the FE analysis of translational motion between the two groups (7.5 ± 1.7 vs. 6.9 ± 2.1 *p* = 0.17). As shown in Table 3, the sitting radiograph revealed the highest kyphotic angle both in Group S and Group M. The lumbar SA in Group S with a sitting position was significantly higher than that in Group M (-5.2 ± 3.7 vs. -1.3 ± 4.9; *p* < 0.001). Compared with Group M, Group S demonstrated significantly lower angular motion in the N-sMR analysis (7.5 ± 2.3 vs. 9.3 ± 3.5; *p* = 0.007), but there was no significant difference in the FE analysis between the two groups (6.7 ± 2.6 vs. 7.7 ± 3.4; *p* = 0.14) (Table 3).

**Table 3:** Measurements of slip percentage and Slip angle via N-U, F-E and N-sMR methods

	**Group S**	**Group M**	***P* Value**
**Slip percentage (%)**			
sitting	24.1 ± 6.3	19.6 ± 5.7	0.002^*^
upright	18.1 ± 3.4	17.4 ± 3.7	0.39
flexion	19.3 ± 4.3	18.9 ± 4.8	0.70
extension	11.7 ± 3.9	11.9 ± 4.1	0.83
Supine MRI	10.3 ± 3.2	11.4 ± 3.8	0.17
Sagittal translation in N-U **(%)**	5.9 ± 2.3	2.1 ± 0.9	< 0.001^*^
Sagittal translation in F-E **(%)**	7.5 ± 1.7	6.9 ± 2.1	0.16
Sagittal translation in N-sMR **(%)**	13.7 ± 4.3	8.1 ± 3.7	< 0.001^*^
**Slip angle** (°)			
sitting	-5.2 ± 3.7	-1.3 ± 4.9	< 0.001^*^
upright	3.7 ± 2.8	8.4 ± 4.3	< 0.001^*^
flexion	-1.9 ± 5.4	1.6 ± 2.4	< 0.001^*^
extension	4.8 ± 3.1	9.3 ± 4.5	< 0.001^*^
Supine MRI	2.3 ± 1.9	7.9 ± 3.7	< 0.001^*^
Sagittal angulation in N-U (°)	8.9 ± 2.8	9.7 ± 3.1	0.24
Sagittal angulation in F-E (°)	6.7 ± 2.6	7.7 ± 3.4	0.14
Sagittal angulation in N-sMR (°)	7.5 ± 2.3	9.3 ± 3.5	0.007^*^

^*^Statistically significant between the group S and group M

### Comparison of the ability to identify instability via NU, FE and N-sMR methods

Translational ≥ motion 8% was considered the criterion of lumbar instability. In Group S, instability was recognized in 83.9% (26 of 31) of patients by N-sMR but only in 22.6% (7 of 31) of patients by FE and in 25.8% (8 of 31) of patients by NU (*P* < 0.001^*^); therefore, the instability of as many as 61.3% (19/31) of patients identified by N-sMR was missed by FE, and the instability of as many as 58.1% (18/31) of patients identified by N-sMR was missed by NU. In Group M, the ability to identify instability was not significantly different between NU, FE and N-sMR (27.6% vs. 31.9% vs. 23.4%, *P* = 0.73). When using the criterion of an angular motion ≥ 10°, instability was recognized in more patients by N-sMR than by FE and NU in Group S (90.3% vs. 22.6% vs. 32.3%, respectively, *P* < 0.001); however, in Group M, no significant difference was observed between N-sMR, FE and NU (29.8% vs. 38.3% vs. 34.0%, respectively, *P* = 0.75). Taken together, a significantly higher incidence of instability was identified in Group S than in Group M (90.3% vs. 38.3%, *P* < 0.001) (Table 4).

**Table 4:** Ability to identify “instability” using N-U, F-E and N-sMR methods

	**N-U, n (%)**	**F-E, n (%)**	**N-sMR, n (%)**	***P* Value**
Translational motion > 8% (translational instability)				
Group S	8(25.8%)	7(22.6%)	26(83.9%)	< 0.001^*^
Group M	13(27.6%)	15(31.9%)	11(23.4%)	0.73
Angular motion ≥ 10° (angular instability)				
Group S	10(32.3%)	7(22.6%)	28(90.3%)	< 0.001^*^
Group M	16(34.0%)	18(38.3%)	14(29.8%)	0.75

^*^Statistically significant between the group S and group M

## Discussion

Objective functional impairment (OFI) is now a widely accepted dimension for evaluating the basic health conditions of patients with various diseases and further providing useful reference data for clinical practice and/or surgical strategy making [7, 16]. Specifically, in spinal pathology, the five-repetition motorized treadmill test (MTT), TUG, 6MWT, and 5R-STS test were employed to evaluate the OFI [16, 17]. Among the aforementioned tests, 5R-STS was validated as the most appropriate test for OFI in patients with degenerative pathologies of the lumbar spine. The 5R-STS test has successfully identified a distinct subset of patients with functional impairment by using the time threshold of 5R-STS time period > 10.4 s [16]. Additionally, there is a strong association between VAS-back pain and OFI as determined by 5R-STS testing. In our study cohort, specifically for patients with DLS, as many as 49 (62.8%) patients were identified with OFI as tested by 5R-STS. However, the association of the positive sign of 5R-STS with clinical symptoms as well as imaging manifestations is unclear. In addition, the potential value of the 5R-STS for OFI in patients with DLS has not been studied. Understanding the potential factors influencing the performance of the 5R-STS test is crucial to determining the reasons why the patients suffer from OFI.

In the current study, patients with positive signs of 5R-STS complained of a higher degree of back pain when bending over to tie shoes, climbing stairs and rising from a chair. Notably, the symptoms of most patients were triggered by movement, including lumbar spinal motions. DLS is a heterogeneous disease that exhibits a large diversity of pain features and precipitating factors. Based on an extensive systematic review of the literature, segmental instability in DLS may play an important role in triggering pain [1, 11, 12, 14, 21]. In our previous study, DLS with a kyphotic configuration was significantly associated with segmental instability and is involved in motions related to low back pain [2]. Additionally, the existence of lumbar lateral instability was considered a main reason for coronal movement-aggravated pain. Staartjes et al. [16]. revealed that performance on the 5R-STS test is affected by demographic factors, including age, height, weight, and BMI. However, the findings of our study are not consistent with the results mentioned above. There was no significant difference in age, sex or BMI between Group S and Group M. However, there is no relevant evidence to clarify the mechanisms of low back pain triggered by the 5R-STS test.

The maximum forward olisthesis of patients with positive signs of 5R-STS was observed in the sitting position, and the minimum olisthesis was observed in the supine position. However, the upright flexion position failed to reveal the maximum forward olisthesis. The role of extension radiography and supine sagittal MR images in reducing forward slippage has been studied, and it is in accordance with a previous study [14]. The reasons why the highest forward slippage occurs in the sitting position are as follows. First, sitting is a normal position with physical weightbearing, and the decreased paraspinal muscle tension in the sitting position may reduce abnormal stress and alleviate low back pain. Second, the increase in the diameter of the spinal canal and nerve foramen in the sitting position may alleviate the clinical symptoms related to neurogenic claudication in patients with DLS. Thus, compared with the traditional standing flexion position, the normal sitting position has the potential to reveal the maximum forward olisthesis. In our study, we found that the sitting radiograph demonstrated a higher slip percentage than the upright standing radiograph (24.1 ± 6.3 vs. 18.1 ± 3.4; *p* < 0.001*) or standing flexion radiograph (24.1 ± 6.3 vs. 19.3 ± 4.3; *p* = 0.001*), which is consistent with the previous study of Zhou [14]. Theoretically, radiographs obtained from sitting positions provide a more effective evaluation of lumbar segmental motion in patients with DLS. However, the article did not identify the relationship between the positive sign of the 5R-STS and pain from sitting to standing or standing to sitting.

Our results also revealed that patients with positive signs of 5R-STS demonstrated the highest translational motion in the N-sMR analysis. Although there are multiple diagnostic modalities in the evaluation of lumbar instability, including FE radiographs and the combination of sagittal CT and MRI, controversy persists for determining the most useful diagnostic modalities for patients with DLS in certain clinical scenarios. In our previous study, the combination of standing upright radiographs and supine MRI was shown to be superior to traditional standing flexion–extension radiographs in measuring translational motion and identifying instability for DLS patients with low back pain and a kyphotic configuration at the slip level [2]. Another study also suggested that upright left and right bending radiographs are more suitable for evaluating lumbar mobility in DLS patients with local coronal instability [15]. As in the current study, N-sMR is an effective radiographic method for evaluating lumbar instability in a distinct subgroup of patients with positive signs of 5R-STS. Furthermore, our results explained the mechanism of low back pain triggered by the sit-to-stand and stand-to-sit motions from a radiographic perspective.

In our study, DLS patients with positive signs of 5R-STS had a greatly kyphotic slip angle in the sitting position. Our results are consistent with previous studies and further indicate that the kyphotic angle at slippage is a sign of segmental instability in the sitting position. When patients were sitting, the sagittal vertical axis moved forward, and the compressive stresses were significantly concentrated on the anterior column. From a biomechanical perspective of the spine, the capacity of the functional spinal unit to withstand shear forces greatly depends on the turgor pressure originating from the healthy intervertebral disc. For patients with insufficient anterior column support, the turgor pressure of the disc decreases, consequently affecting the disc with regard to anterior shear forces and subsequently resulting in vertical instability. In a biomechanical study by Luk et al. [21], there was a significant relationship between the intervertebral kyphotic slip angle and “vertical instability”. However, it is worth mentioning that “vertical instability” cannot be measured with traditional flexion–extension radiographs. As demonstrated in our study, for patients with positive signs of 5R-STS, FE failed to detect real angular motion, but N-sMR effectively revealed a higher degree of sagittal angulation.

A positive performance of the 5R-STS test is an indicator of OFI, and our results provide direct radiographic evidence to clarify their relationship. With a translational motion ≥ 8% or angular motion ≥ 10° as the instability criterion, in Group S, as many as 83.9% (26 out of 31) of patients with positive signs of 5R-STS were detected to have lumbar instability by N-sMR. Our study reveals the possible reasons why low back pain is triggered by the daily sit-to-stand movements. Additionally, our findings may be clinically relevant when offering counseling to patients with DLS regarding the preoperative assessment. The 5R-STS test is a simple and effective tool for detecting OFI in patients with DLS [16]. Patients who present with a greater degree of OFI generally achieve a relatively higher surgical benefit. The evaluation of OFI by the 5R-STS test can provide surgeons with a more detailed and comprehensive assessment of a patient’s impaired function status and provide more useful information for surgical intervention. Thus, in our clinical practice, for patients with positive signs of 5R-STS, we commonly prescribe N-sMR in detecting lumbar instability of DLS.

There are several limitations in this study that could be addressed in future research. First, this study enrolled a small sample size of patients with DLS at our single center. However, the enrolled patients represented classical lumbar spondylolisthesis, and patients received a detailed and comprehensive assessment of a patient’s impaired function status to eliminate misclassification and selection bias. Second, the effective outcomes of the 5R-STS test depend on the examiner’s exact instructions and on the patient’s cooperation and can lead to different results from test to test.

## Conclusion

The main cause of low back pain triggered by sit-to-stand movements is related to lumbar spine instability, and the positive performance of the 5R-STS presented in patients with DLS can be considered a sign of lumbar instability. The combination of normal sitting and supine positions has clinical value for evaluating translational motion and angular motion, and N-sMR is the most clinically relevant modality for measuring sagittal translation and identifying instability in DLS patients with positive performance of 5R-STS.

## Data Availability

The datasets generated and/or analysed during the current study are not publicly available due patients privacy but are available from the corresponding author on reasonable request.

## Abbreviations

DLS: Degenerative lumbar spondylolisthesis
5R-STStest: Five-repetition sit-to-stand test
MRI: Magnetic Resonance Imaging
ODI: Oswestry disability index
VAS: Visual analog scale
PROs: Patient‑reported outcomes
SP: Slip percentage
SA: Slip angle
U: Upright standing radiograph
N: Normal sitting radiograph
F: Flexion radiograph
E: Extension radiograph
sMR: Supine sagittal MR image
NU: Normal sitting and upright standing radiograph
FE: Flexion and extension radiograph
N-sMR: Normal sitting radiograph and supine sagittal MR image
L4: Lumbar 4
L5: Lumbar 5
L4/5: Lumbar 4/5

## References

[1] Gautschi OP, Smoll NR, Corniola MV, Joswig H, Chau I, Hildebrandt G (2016). Validity and Reliability of a Measurement of Objective Functional Impairment in Lumbar Degenerative Disc Disease. Neurosurgery.

[2] Chen X, Zhou Q, Xu L, Chen Z, Zhu Z, Li S (2018). Does kyphotic configuration on upright lateral radiograph correlate with instability in patients with degenerative lumbar spondylolisthesis?. Clin Neurol Neurosurg.

[3] Farshad-Amacker NA, Farshad M, Winklehner A, Andreisek G (2015). MR imaging of degenerative disc disease. Eur J Radiol.

[4] Chen X, Xu L, Qiu Y, Chen Z, Zhou Q, Li S (2018). Higher Improvement in Patient-Reported Outcomes Can Be Achieved After Transforaminal Lumbar Interbody Fusion for Clinical and Radiographic Degenerative Spondylolisthesis Classification Type D Degenerative Lumbar Spondylolisthesis. World Neurosurgery.

[5] Landi A, Gregori F, Mancarella C, Maiola V, Maccari E, Marotta N (2015). Lumbar spinal degenerative “microinstability”: hype or hope? Proposal of a new classification to detect it and to assess surgical treatment. Eur Spine J.

[6] Weinstein JN, Tosteson ANA, Blood E, Herkowitz H, Boden SD, Berven S. Surgical versus Nonsurgical Therapy for Lumbar Spinal Stenosis. N Engl J Med. 2008;358:794–810. 10.1056/NEJMoa0707136 PMC2576513 18287602

[7] Klukowska AM, Schröder ML, Stienen MN, Staartjes VE (2020). Objective functional impairment in lumbar degenerative disease: concurrent validity of the baseline severity stratification for the five-repetition sit-to-stand test. J Neurosurg Spine.

[8] Atlas SJ, Delitto A (2006). Spinal Stenosis: Surgical versus Nonsurgical Treatment. Clin Orthop Relat Res.

[9] Staartjes VE, Klukowska AM, Schröder ML (2020). Association of maximum back and leg pain severity with objective functional impairment as assessed by five-repetition sit-to-stand testing: analysis of two prospective studies. Neurosurg Rev.

[10] Stienen MN, Ho AL, Staartjes VE, Maldaner N, Veeravagu A, Desai A (2019). Objective measures of functional impairment for degenerative diseases of the lumbar spine: a systematic review of the literature. Spine J.

[11] Iguchi T, Ozaki T, Chin T, Tsumura N, Kanemura A, Kasahara K (2011). Intimate relationship between instability and degenerative signs at L4/5 segment examined by flexion–extension radiography. Eur Spine J.

[12] Tarpada SP, Cho W, Chen F, Amorosa LF (2018). Utility of Supine Lateral Radiographs for Assessment of Lumbar Segmental Instability in Degenerative Lumbar Spondylolisthesis. Spine.

[13] Huang K-Y, Lin R-M, Lee Y-L, Li J-D (2009). Factors affecting disability and physical function in degenerative lumbar spondylolisthesis of L4–5: evaluation with axially loaded MRI. Eur Spine J.

[14] Zhou Q, Sun X, Chen X, Xu L, Qian B, Zhu Z (2021). Utility of Natural Sitting Lateral Radiograph in the Diagnosis of Segmental Instability for Patients with Degenerative Lumbar Spondylolisthesis. Clin Orthop Relat Res.

[15] Wang XW, Chen X, Fu Y, Chen X, Zhang F, Cai HP, Ge C, Zhang WZ. Analysis of lumbar lateral instability on upright left and right bending radiographs in symptomatic patients with degenerative lumbar spondylolisthesis. BMC Musculoskelet Disord. 2022;23(1):59. 10.1186/s12891-022-05017-1. 10.1186/s12891-022-05017-1 PMC8764856 35039039

[16] Staartjes VE, Schröder ML (2018). The five-repetition sit-to-stand test: evaluation of a simple and objective tool for the assessment of degenerative pathologies of the lumbar spine. J Neurosurg Spine.

[17] Jones SE, Kon SSC, Canavan JL, Patel MS, Clark AL, Nolan CM (2013). The five-repetition sit-to-stand test as a functional outcome measure in COPD. Thorax.

[18] Hey HWD, Lau ET-C, Lim J-L, Choong DA-W, Tan C-S, Liu GK-P (2017). Slump sitting X-ray of the lumbar spine is superior to the conventional flexion view in assessing lumbar spine instability. Spine J..

[19] Telli H, Hüner B, Kuru Ö (2020). Determination of the Prevalence From Clinical Diagnosis of Sacroiliac Joint Dysfunction in Patients With Lumbar Disc Hernia and an Evaluation of the Effect of This Combination on Pain and Quality of Life. Spine.

[20] Takahashi T, Hanakita J, Watanabe M, Kawaoka T, Takebe N, Kitahara T (2014). Lumbar Alignment and Clinical Outcome after Single Level Asymmetrical Transforaminal Lumbar Interbody Fusion for Degenerative Spondylolisthesis with Local Coronal Imbalance. Neurol Med Chir..

[21] Luk KDK, Chow DHK, Holmes A (2003). Vertical Instability in Spondylolisthesis: A Traction Radiographic Assessment Technique and the Principle of Management. Spine.

